# Effect of intervention on the control of Highly Pathogenic Avian Influenza in Nigeria

**Published:** 2012-09-19

**Authors:** Agnes Tinuke Oladokun, Clement Adebajo Meseko, Edelokun Ighodalo, Benshak John, Pius Stephen Ekong

**Affiliations:** 1 Viral Research Division, National Veterinary Research Institute, Vom, Nigeria; 2 Central Diagnostic Laboratory, National Veterinary Research Institute, Vom, Nigeria; 3 Applied Biotechnology, National Veterinary Research Institute, Vom, Nigeria; 4 Epidemiology Section, National Veterinary Research Institute, Vom, Nigeria

**Keywords:** Highly Pathogenic Avian Influenza, H5N1, intervention, control, surveillance, Nigeria

## Abstract

**Introduction:**

The advent of HPAI in Nigeria was a traumatic experience for the poultry industry. Wealth and resources were lost to the ravages of the virus. The Government of Nigeria with the support of International donor agencies came up with a policy for the prevention of spread of the disease leading to the eventual control and eradication of the virus in Nigeria. The various measures implemented in the control of the outbreaks, and their effects on eradication of the virus in the country are highlighted.

**Methods:**

Using combined data from passive and active surveillance for HPAI in poultry farms, wetlands and live bird markets in Nigeria during 2006 – 2009, with laboratory diagnostic findings, we describe the characteristics of the control strategies implemented. The control measures include immediate reports of suspected outbreaks, prompt laboratory confirmation and rapid modified stamping out with compensations paid to affected farmers. Decontamination of infected farm premises, re-organization of live bird market were carried out, and bio security measures put in place before re-stocking.

**Results:**

Three years following initial outbreak, the number of laboratory confirmed cases drastically reduced from 140 in 2006 and 160 in 2007 to only 2 cases of field outbreak in 2008. Only one case of human infection was documented during the period and no field outbreak or detection by surveillance was reported throughout 2009 and 2010.

**Conclusion:**

The measures employed by the Government through its agencies in the control of HPAI in Nigeria brought the incidence of the disease to naught

## Introduction

Influenza viruses are found predominantly in waterfowls [1, 2]. The original virus that spread to humans in 1997 was first detected in Guangdong, China in 1996. This H5N1 virus was eradicated by the culling of all domestic poultry in Hong Kong. Different reassortant of this virus however continued to emerge and spread to different regions [3].

Highly Pathogenic Avian Influenza caused respiratory disease and deaths in poultry birds and in farmers and consumers that were inappropriately exposed to aerosols generated from handling chickens. The virus appears most threatening, acquiring unprecedented capacity to cause high proportion of death in birds and to cause death and be transmitted among wild species, including domestic cats [4]. The fear is that the magnitude of the virulence and pathogenicity of the virus is yet to manifest until a pandemic strain evolves.

The initial incidence of the disease in Hong Kong, 1997 was prelude to the 2003 sporadic outbreaks in Asia. This was the precursor of the virus that was detected in Nigeria which also spread to other African countries like Egypt, Togo and Ivory- coast [5].

Nigeria recorded the first outbreak of Highly Pathogenic Avian Influenza (HPAI) in February 2006 in a commercial poultry farm in Northern Nigeria. The outbreak was not sudden on the country, because of the situation with regards to HPAI in the global community, the Federal Government already had an emergency preparedness plan in place which to a great extent paved way for the initial control strategy. WHO had recommended all countries at risk to develop and update their influenza pandemic preparedness plan. Nigerian Pandemic Preparedness and Action Plan for Avian Influenza were produced in response to this international request, which is also in consonance with the national philosophy, policy and goal to safeguard the health and socio-economic well-being of all Nigerians.

The source of the initial outbreak in Nigeria is still not clear; international trade in birds or wild migratory birds could be responsible [6]. The two routes of introduction transmission may obviously be involved as later evidence suggests [7] and the virus subsequently spread to all the agro-ecological regions of the country killing over 1.2 million domesticated birds and many more culled to stop its spread [5]. This study highlights the various control measures implemented in the control of the outbreaks of HPAI virus in Nigeria, and their effect on the eradication of the virus in the country.

## Methods

In January 2006, a report of an outbreak of an unknown disease at Sambawa farms Jaji, Kaduna state was received by the National Veterinary Research Institute, Vom. Following laboratory analyses, the virus isolated was typed as Influenza A (Avian Influenza), which was subsequently confirmed by the OIE, FAO and National Reference Laboratory for Newcastle disease and Avian Influenza viruses in Padova Italy, as HPAI (H5N1) based on the amino acid sequences (PQGERRRKKRGLFG) at the cleavage site of Haemagglutinin gene [8]. The result of the confirmation of the diagnosis and sub typing was received on February 6, 2006 and a public and formal notification of the outbreak was made on February 8, 2006. The Federal Government instituted immediate control and preventive measures through modified depopulation of affected farms. The disease was subsequently reported and confirmed in 27 states and the FCT.

In the implementation of the control strategies, the committee of experts established by the Federal Ministry of Agriculture and Rural Development put in place measures to prevent the spread of the disease in the country following the initial outbreak. Consequently the following actions were taken by the Veterinary authority: Total ban on importation of poultry and poultry products; National Quarantine Services and other related agencies were put on alert to monitor national borders and interstate control posts; Intensification of surveillance at designated surveillance points, live bird markets, International and Interstate control posts; Laboratory capacity building/training and guidelines for proper disinfection of all equipment, premises and infected areas with clear procedures for the sanitary disposal of carcasses.

### Sampling

As part of the set out agenda in the plan for the control of HPAI, poultry farmers and rural flock holders were enlightened on the need to report suspicions of the disease to the nearest Veterinary authority, who had been mobilized to collect samples promptly and deliver it under cold chain to the National Veterinary Research Institute, Vom at no cost to the farmer in a passive surveillance strategy. This method of sample collection was strengthened when the Avian Influenza Control Programme − Animal Health (AICP-AH) was structured with funding from the World Bank. Hence each state has a desk officer whose responsibility is to investigate field suspicions, collect appropriate specimen and send to the National laboratory.

**Figure 1:**
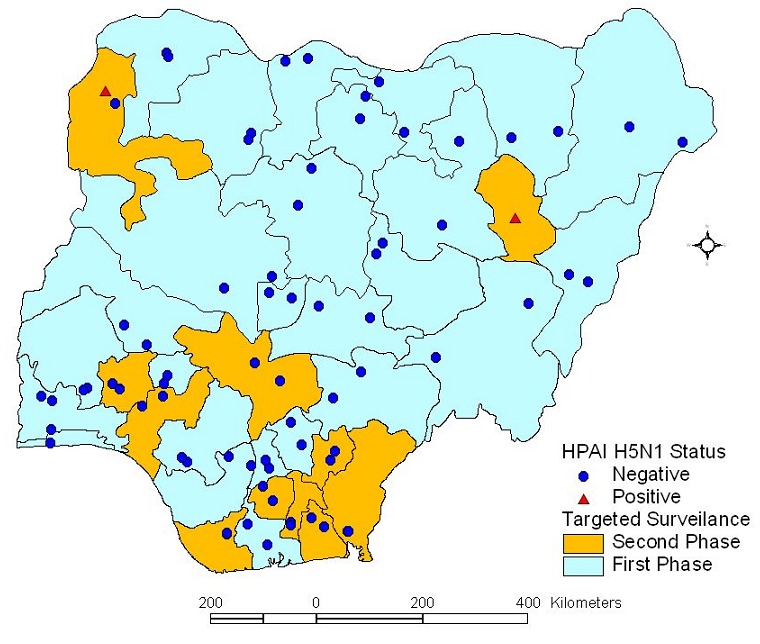
Map of Nigeria showing the surveillance points and Highly Pathogenic Avian Influenza H5N1 status during the targeted HPAI surveillance in Nigeria, 2006-2008

In furtherance, active surveillance programme was organized thematically. The first being a random survey in selected geo reference points across the country. Five hundred and sixty points were identified and tracheal swabs, cloacal swabs and sera samples were collected from various avian species in virus transport medium and transported in cold chain to the laboratory for analysis. This was followed by targeted surveillance, the first phase was carried out in two live bird markets (LBM) each in 25 States that had confirmed cases of HPAI, while the second phase was carried out in States where infections was not earlier reported (Figure 1). In both cases, sample collection was repeated in the same LBM at two week intervals. Samples collected were similar to those collected in the nationwide surveillance, but in addition, carcasses of sick and moribund birds were included in the samples sent to the laboratory. Specimen was collected from 25 birds in two selected LBMs in each of the 11 states.

### Laboratory Analyses

Avian carcasses were pathologically examined for evidence of HPAI. Whole carcasses with the classical avian influenza signs of severe haemorrhages, swollen and bluish wattles and combs, had their cloacae swabbed. Faecal materials or cloacal mucosa debris were used for rapid antigen detection. Parenchymatous tissues were homogenized in antibiotic solution, the suspension was spun and the supernatant inoculated into the allantoic cavities of 9 to 11-day old chicken embryonating eggs, these were then incubated for 7 days for the first passage. Embryo mortality is monitored and dead ones were chilled for a minimum of 1 hour, thereafter the allantoic fluid is harvested for rapid haemagglutination test. Specimens that are positive were typed for Influenza A antigen by Agar Gel Immuno Diffusion (AGID) test and Haemagglutination Inhibition test with H5 monospecific antiserum. Molecular methods were then used to identify both the M and H5 genes by Reverse-transcriptase polymerase chain reaction (RT-PCR). The swabs were screened for influenza A by RT-PCR. This involved initial RNA extraction of the virus using Qiagen extraction kit, followed by amplification of the nucleic acid in a Thermo cycler by one step RT-PCR reaction. Detection of the amplified gene was done by gel electrophoresis, and the product was thereafter captured in gel documentation system. Swab samples that were positive by RT-PCR were subjected to virus isolation in chicken embryonating eggs. Further molecular characterization and sequencing of all isolates or amplified genes were carried out at the OIE reference laboratory for Avian influenza and Newcastle disease in Padova, Italy.

### Control Measures

When samples were taken on the field, they were immediately dispatched in cold chain and received at the laboratory. These were analyzed as described above and results were immediately communicated to the Federal Livestock Department who in turn embarked on prompt depopulation and decontamination of farms with positive results. Such farmers were then paid compensation based on the culled birds. Table 1 shows a summary of the distribution of states affected by the HPAI H5N1, number of birds depopulated and total compensation paid to affected farmers from February 2007 to January 2008.

**Table 1:** Distribution of states affected by the HPAI H5N1, number of birds depopulated and total compensation paid to affected farmers from February 2007 to January 2008

State	Affected LGAs	No. of Beneficiaries	Depopulated 2006	Depopulated 2007	Total Depopulated	Total Compensation (Naira)
Adamawa	4	211	0	14,974	14,974	3,381,200
Anambra	4	5	1,465	4,111	5,576	5,401,170
Bauchi	5	50	117,042	4,369	121,411	36,726,625
Benue	1	0	0	0	0	0
Borno	2	8	256	890	1,146	1,028,795
Delta	1	1	0	1,784	1,784	1,294,500
Edo	4	5	30	4,004	4,034	4,097,700
Ekiti	1	1	0	1,088	1,088	979,200
Enugu	2	0	0	0	0	0
FCT	3	609	206	28,287	28,493	11,415,100
Jigawa	2	4	0	12,965	12,965	17,923,145
Kaduna	8	113	47,378	45,411	92,789	69,114,950
Kano	17	147	229,781	235,053	464,834	129,101,480
Katsina	6	35	4,905	87,300	92,205	120,586,345
Kwara	1	2	4,610	0	4,610	1,152,500
Lagos	9	89	50,985	13,234	64,219	25,857,740
Nasarawa	5	895	22,023	1,062	23,085	12,659,770
Niger	1	1	0	27	27	210,600
Ogun	6	25	88,302	54,893	143,195	72,587,325
Oyo	2	1	0	11,482	11,482	10,649,800
Plateau	2	33	62,916	48,011	110,927	49,211,380
Sokoto	2	2	0	29,542	29,542	42,571,295
Rivers	1	376	12,446	0	12,446	2,144,000
Taraba	2	74	1,018	0	1,018	362,710
Yobe	3	44	0	5,846	5,846	1,665,500
Zamfara	2	2	0	2,649	2,649	2,955,050
Total	97	2,733	643,363	1,250,345	1,893,708	623,077,880
Summary	FGoN	1,712				172,797,590
	WB	1,021				450,280,290
	**Total**	**2,733**				**623,077,880**

FGoN – Federal Government of Nigeria; WB – World Bank; Source: FDL&PCS of the Federal Ministry of Agriculture and Water Resources, Nigeria

### Restriction on bird movement

Restriction was imposed on the movement of live poultry in the country- inter-state, intra-state movement of live poultry from areas affected and transportation of poultry in passenger vehicles. Task forces were set up by different states to manage and prevent the spread of the outbreak.

### Public awareness creation

Public enlightenment and awareness programs on HPAI identification, diagnosis, prevention and controls involving all stakeholders were carried out in form of trainings, workshops, seminars, television and radio jingles in all states of the country. Farmers were educated on the need to observe strict bio-security measures. The public was advised on the need to thoroughly wash their hands after handling birds and to properly cook bird meat before consumption. The public was equally educated on the need to see a physician if they experience influenza like symptoms.

### Depopulation and decontamination

Infected farms were depopulated by trained personnel who buried the affected birds in dug-out pits. Such premises were then decontaminated using 1% Virkon^®^. Bio security measures such as fallowing of farms for 3 months before restocking, movement control, foot dipping, perimeter fencing of farms were put in place to control spread of infection from farm to farm.

### Re-organization of poultry farms

As a means of control and identification, registration of poultry farms with over 200 birds was made compulsory in order to keep records of poultry population and contingency planning and intervention. Farmers were also educated on the danger of keeping multispecies and multiage birds, and interaction of free range with the intensively raised birds as related to the epidemiology of the disease.

### Re-organization of LBM

One major identified risk area is live bird markets where sometimes sick birds are brought from farm and vice versa. The following control measures were instituted to contain spread of HPAI through this means: Active surveillance of the disease in live bird markets - Sera, tracheal and cloacal swabs were collected and sent to NVRI Vom for laboratory analysis; Weekly fumigation and decontamination of these live bird markets; Training of personnel in disease recognition and actions required in possible outbreak; and Public enlightenment through workshop/seminars and media publicity for the farmers and other stakeholders on the importance of bio security.

### Enforcement of good laboratory practices

To prevent human exposure and environmental contamination from the submitted specimen, all laboratory procedures were carried out under strict bio containment conditions.

## Results

**Figure 2:**
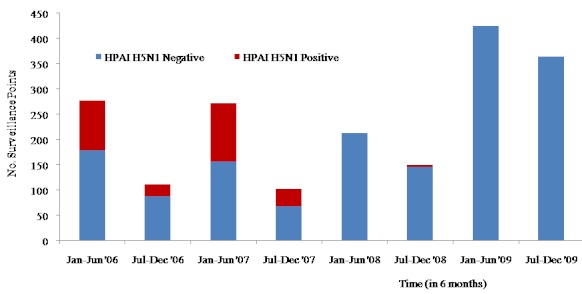
Distribution of the HPAI H5N1 positive and negative samples collected during Highly Pathogenic Avian Influenza surveillance in Nigeria, 2006-2009

Three years following initial outbreak, the number of laboratory confirmed cases drastically reduced from 140 in 2006 and 160 in 2007 to only 2 cases of field outbreak in 2008. Only one case of human infection was documented during the period and no field outbreak or detection by surveillance was reported throughout 2009 and 2010 (Figure 2).

## Discussion

Through passive surveillance, 890, 970 and 450 samples were respectively received at the laboratory in 2006, 2007 and 2008 for HPAI. The nationwide active surveillance yielded 11,334 samples, while targeted surveillance in live bird markets in infected and non infected states yielded 13,884 and 5,807 samples respectively. No virus isolation was recorded during the nationwide surveillance. However, targeted surveillance in live bird market detected the presence of the virus.

The 1-5 kilometres radius of depopulation could not be implemented because of the adverse effect it would have on the economic and livelihood particularly in the rural communities. The stamping out were carried out by trained personnel to avoid spreading infection and human exposure using personal protective equipments (PPE). Affected farmers were compensated initially at N250 per adult bird, but when it was observed that this was not sufficient and farmers may not report outbreaks, the amount was reviewed upwards to N1200 per adult bird. This discouraged concealment of outbreaks and chances of spread of infection.

## Conclusion

In controlling HPAI, the Federal Government of Nigeria with the assistance of donor agencies launched several intervention programmes. Chief among these are surveillance in poultry farms, wetlands, live bird markets and rural free range birds. All these were combined with public awareness on disease recognition, report of suspicions, prompt sample collection and delivery to the laboratory. The principle and practice of biosecurity was also emphasized with the re-organization and restructuring of live bird markets. The synergy of prompt laboratory confirmation of cases and rapid depopulation and decontamination on the field impacted positively on the control of the disease in the country without the use of vaccination. We therefore recommend this as sustainable measure in the control of HPAI in resource poor countries of Africa.
